# Return to work experiences of patients treated with stem cell transplantation for a hematologic malignancy

**DOI:** 10.1007/s00520-018-4596-0

**Published:** 2018-12-27

**Authors:** S. Persoon, L. M. Buffart, M. J. M. Chinapaw, F. Nollet, M. H. Frings-Dresen, S. Koning, M. J. Kersten, S. J. Tamminga

**Affiliations:** 1 Amsterdam UMC, University of Amsterdam, Rehabilitation Medicine, Amsterdam Movement Sciences institute, Amsterdam, The Netherlands; 2 Amsterdam UMC, Vrije Universiteit Amsterdam, Department of Epidemiology and Biostatistics, Amsterdam Public Health research institute, Amsterdam, The Netherlands; 3 Amsterdam UMC, Vrije Universiteit Amsterdam, Department of Medical Oncology, Cancer Center Amsterdam, Amsterdam, The Netherlands; 4 Exercise Medicine Research Institute, Edith Cowan University, Joondalup, Australia; 5 Amsterdam UMC, Vrije Universiteit Amsterdam, Department of Public and Occupational Health, Amsterdam Public Health research institute, Amsterdam, The Netherlands; 6 Amsterdam UMC, University of Amsterdam, Coronel Institute of Occupational Health, Amsterdam, The Netherlands; 7 Amsterdam UMC, University of Amsterdam, Department of Hematology, Amsterdam, The Netherlands

**Keywords:** Hematopoietic stem cell transplantation, Hematologic malignancy, Return to work (RTW), Qualitative research

## Abstract

**Purpose:**

This qualitative study aimed to identify hematopoietic stem cell transplantation (HSCT) survivors’ (1) work perceptions; (2) barriers to and facilitators of return to work (RTW); and (3) possible solutions to improve RTW.

**Method:**

Fifteen patients treated with HSCT 1–5 years ago participated in face-to-face semi-structured interviews. Interviews were analyzed following the steps of thematic content analyses.

**Results:**

RTW was often characterized as a complex and prolonged trajectory, and it was frequently incomplete in working hours, tasks, and/or responsibilities. Work perceptions varied between patients; most valued work as positive, but some also reported a decline in work capacity and/or in importance. Perceived barriers included the duration and side effects of cancer treatment, the presence of comorbidity and poor health before diagnosis, having difficulties commuting and doing household tasks. Perceived facilitators were financial incentives, keeping in touch with the workplace, support of other patients and family, and looking after one’s health. Proposed solutions to improve RTW included discussing RTW at the hospital, enhanced employer support, improved accessibility of rehabilitation programs, and more information about the consequences of being sick-listed.

**Conclusions:**

Many HSCT survivors value work as important and they are motivated to RTW. Insight in work perceptions, RTW barriers, and solutions might help researchers, healthcare professionals, and employers to develop and/or tailor individualized multidisciplinary care to facilitate RTW.

## Introduction

Hematopoietic stem cell transplantation (HSCT) has improved the survival of patients with various hematologic malignancies [1]. However, this aggressive type of treatment is frequently associated with short- and long-term medical complications [1–3], physical and psychological symptoms [2, 4], and reduced quality of life (QoL) [4, 5]. Not surprisingly, a substantial proportion of HSCT survivors does not, or only partially, return to work (RTW) [6–13]. A previous study found for instance that only 36% of the HSCT survivors who worked full-time before transplant had returned to full-time work 1 year after transplant [8], and this proportion had increased to 60% after 5 years [8]. The same study showed that 36% of the HSCT survivors working full-time 3 years after transplant reported ongoing work-related limitations with a diminished ability to accomplish as much at work compared with the pre-illness situation [8]. Similarly, in a Swedish study, approximately 40% of the patients that were on average 10 years post-transplant reported a reduced physical work capacity [9].

Improving sustainable RTW might improve Qol [14, 15]. Previous reviews in which the experiences with work of patients with various types of cancer were evaluated reported that (return to) work may restore a sense of normalcy and/or a sense of the former self, and provide financial security, distraction [15, 16], and social interaction [16].

Currently, only limited data are available on the attitudes toward and experiences with RTW of HSCT survivors. Recently, an American qualitative study evaluated the long-term experiences with work and finances among patients treated with autologous stem cell transplantation [17]. Notable were the reported financial challenges and health insurance difficulties [17]. Furthermore, a previous Australian qualitative study explored the survivorship and RTW experiences of patients treated for hematologic malignancies, of whom half were treated with HSCT [18]. This study focused on patients who faced major problems with returning to work, the facilitators of successful RTW and recommendations for supportive care organizations, and to a lesser extent on perceived problems while being at work [18]. The findings of these studies, notwithstanding their importance, may not be generalizable to other countries given the country-specific laws and regulations. In the Netherlands, both patients and employers are responsible for RTW. During the first 2 years of sick-leave, patients cannot be fired because of illness and they receive at least 70% of their salary. Subsequently, patients are assessed for disability pension [19].

Given the scarcity of literature on this topic, more research on HSCT survivors’ experiences with their RTW is warranted. This will aid the development of interventions supporting survivors to successfully RTW. Therefore, this qualitative study aimed to identify HSCT survivors’ (1) (return to) work perceptions; (2) barriers to and facilitators of RTW; and (3) possible solutions to improve RTW.

## Methods

We conducted 15 face-to-face semi-structured interviews between March and May 2014 and followed the consolidated criteria for reporting qualitative research (COREQ) [20]. Ethical approval from the Medical Ethics Committee of the Academic Medical Center (AMC) was sought, but this Committee decided that an extensive test on ethical and juridical aspects was unnecessary (W13_033). The Medical Ethical Committee had no objection to the execution of this study.

### Patient recruitment and study procedures

Patients were recruited from the outpatient clinic of the hematology department of an academic hospital using a convenience sampling process. They were eligible for participation if they were: (1) treated with HSCT for a hematologic malignancy 1–5 years earlier; (2) aged between 18 and 60 years at the time of the HSCT; (3) working at least 12 h per week in the year prior to the HSCT; (4) fluent in Dutch; (5) in remission since the HSCT; and (6) having no severe physical or mental comorbidity at time of the interview. Recruitment was stopped after 15 interviews. We determined this sample size a priori based on our experience with reaching data saturation after inclusion of 12 patients in a previous qualitative study regarding barriers to and facilitators of RTW of breast cancer survivors [21], assuming that we would reach data saturation with 10–15 patients.

Potentially eligible patients were informed about the study by their treating hematologist. Hereafter, SK (a female master student) contacted these patients by telephone to re-establish eligibility and willingness to participate. If patients fulfilled the criteria, an appointment for the interview was made. Prior to the interview, patients were asked to fill out a short questionnaire on disease, treatment, and work-related characteristics. The aim of the study, the position of the interviewer, and the guarantee that the data were handled confidentially were discussed with each patient. Hereafter, patients gave their written informed consent.

The face-to-face semi-structured interviews were audio-recorded. Thirteen (87%) patients were interviewed at the outpatient clinic and two at home. Each interview started with open-ended questions followed by more specific in-depth questions on: (1) the diagnosis and treatment and pre-treatment work situation, (2) work perceptions, (3) perceived barriers to and facilitators of RTW, and (4) possible solutions to improve RTW. The topic list was pilot tested in an interview with a patient with multiple myeloma.

The interviewer (SK) summarized all factors mentioned at the end of the interview to check for misunderstandings and completeness.

### Analysis

The MAXQDA (Verbi GmbH, Marburg, Germany version 11) qualitative data analysis software package was used to code the transcripts.

All transcripts were analyzed following a thematic content analysis approach [22]. First, every transcript was read to become familiar with the content of the interview. Second, transcripts were open-coded to identify important answers to the research questions. The labels of the open codes represented the transcript as closely as possible. To be able to analyze different sub-questions separately, labels could be used in multiple classifications.

Third, to identify the work perceptions, the open codes were translated into themes (axial coding) and these themes were divided into four sub-themes (selective coding): RTW motivation, RTW values, feelings about RTW, and perceived work ability. The first three themes were taken from the overview presented by De Jong et al. [23]. For the development of this overview, the experiences and perceptions of working life of employees with a chronic disease as reported in 61 publications had been synthesized and categorized in five categories [23].

Fourth, to comprehensively identify barriers to and facilitators of RTW, the open codes were classified using the International Classification of Functioning, Disability and Health (ICF) model developed by the World Health Organization [24]. The ICF-model enables a comprehensive and transparent analysis, as it describes the interaction between health and disability, body function and structures, activities and participations, and personal and environmental factors [24]. Subsequently, open codes were translated into themes (axial coding) and these themes were translated into the factors of the core set for vocational rehabilitation (selective coding) [25].

Fifth, to identify the possible solutions to improve RTW, open codes were translated into themes. SK performed all five above-mentioned steps. Thereafter, ST repeated steps 3 to 5 of the analyses conducted by SK, with knowledge of the outcome of the analyses done by SK. ST and SP discussed the final themes until consensus was reached.

## Results

Recruitment was stopped after 15 interviews. Before reaching this number, 22 patients had been invited, of whom 7 (32%) did not participate for unknown reasons. 47% of the patients were female, the median age of the patients was 48 years (range 30–59), 33% had a bachelor’s or master’s degree, 73% were married or living together, and the patients were on average 30 months (range 15–57) post-HSCT (Table 1). The median duration of the interviews was 38 (range 13–73) minutes.

**Table 1:** Demographics and clinical characteristics of the patients treated with HSCT

Pt	Age, years	Diagnosis	Type of HSCT	Intensity of conditioning^a,b^	Chronic GVHD^c^	Months since HSCT
1	≥ 45	AML	Allo	Reduced intensity	No	< 24
2	≥ 45	ALL	Allo	Reduced intensity	No	> 24
3	< 45	AML (relapsed)	Allo	Myeloablative	No	< 24
4	< 45	NHL (relapsed)	Allo	Reduced intensity	No	> 24
5	≥ 45	AML	Allo	Reduced intensity	Yes	< 24
6	≥ 45	AML	Allo	Myeloablative	Yes	> 24
7	≥ 45	NK LGL leukemia	Allo	Reduced intensity	No	< 24
8	≥ 45	MM	Auto	–	–	> 24
9	< 45	AML	Allo	Myeloablative	Yes	> 24
10	< 45	AML	Allo	Reduced intensity	Yes	< 24
11	≥ 45	MM	Auto	–	–	< 24
12	< 45	AML	Allo	Myeloablative	No	> 24
13	≥ 45	NHL	Auto	–	–	> 24
14	< 45	T-lymphoblastic lymphoma	Allo	Myeloablative	Yes	> 24
15	≥ 45	NHL (relapsed)	Auto	–	–	< 24

*Abbreviations*: *Pt* patient, *AML* acute myeloid leukemia, *ALL* acute lymphoblastic leukemia, *NK LGL leukemia* natural killer cell large granular lymphocyte leukemia, *NHL* non-Hodgkin lymphoma, *MM* multiple myeloma, *Allo* allogeneic stem cell transplantation, *Auto* autologous stem cell transplantation, *HSCT* hematopoietic stem cell transplantation, *GVHD* graft-versus-host disease

^a^Only applicable to allogeneic stem cell transplantation survivors

^b^Intensity of the preparation regimen given directly before HSCT, a myeloablative conditioning regimen will cause pancytopenia and a stem cell support is needed to restore bone marrow function, while a reduced intensity regimen is less intensive and only causes partial cytopenia

^c^Based on self-report

### Work-related characteristics

Twelve patients (80%) had a permanent employment contract before they were diagnosed with cancer, two (13%) were self-employed, and one (7%) had a temporary employment contract (Table 2).

**Table 2:** Work-related characteristics of the patients treated with HSCT

Pt	Work status before diagnosis				Current occupation (working hours)			RTW and current work characteristics
	Occupation	Years in company before HSCT	Contract	Hours	Occupation	Contract	Hours (contract)/currently working	
1	Office manager	< 5	Perm	36	Disability pension	NA	NA	• Stopped working directly after diagnosis, initial RTW ~ 10 months later (officially sick-listed) • Disability pension started 2 years after diagnoses
2	Specialized traffic controller	≥ 5	Perm	36	Security supervisor	Perm	36/24 (officially sick-listed)	• Stopped working directly after diagnosis, initial RTW between 1.5 and 2 years later • Officially still 100% sick-listed • Assignment to job of lower rank, without irregular shifts • Potential further partial loss of income in near future
3	Sales assistant	< 2	Temp	20	Disability pension	NA	NA	• Stopped working directly after diagnosis (of relapse) • Fixed-term employment contract was not extended
4	Customer relationship manager	< 5	Perm	32	Same	Perm	32/32	• Officially 100% sick-listed after diagnosis, but performed some work tasks during the first treatment period • Did not work in the month succeeding SCT, gradual RTW hereafter • Less and unpleasant job tasks during initial RTW
5	Scientist	≥ 5	Perm	40	Manager	Perm	40/40	• Stopped working directly after diagnosis, initial RTW ~ 7 months later, full RTW more than 1.5 year after diagnosis • Assignment to job of higher rank/promotion
6	Account manager	< 2	Perm	40	Same	Perm	36/0	• Stopped working directly after diagnosis, initial RTW ~ 1 year later, full RTW 2 years after diagnosis • Different job tasks • 1 year after full RTW, new period of sick-leave
7	Financial employee	≥ 5	Perm	40	50% disability pension, 50% same	Perm	40/20	• Recurrent periods of sick-leave in the years before SCT • 6 months after SCT initial RTW • Less challenging job tasks
8	Warehouse employee	≥ 5	Perm	40	Same	Perm	40/40	• Stopped working after diagnosis, initial RTW ~ 8 months later, and full RTW 1 year after diagnosis. • 1 month later, new period of radiation therapy and partly sick-listed • Worked 100% at the time of the interview
9	Office sales worker	≥ 5	Perm	37.5	Disability pension	NA	NA	• Before diagnosis, reintegrating and officially 100% sick-listed because of a shoulder injury • Stopped reintegration trajectory after diagnosis • Disability pension started in month succeeding HSCT
10	Self-employed	Unknown	–	65	Disability pension^a^	NA	NA	• Stopped working after diagnosis, closed his company • Plans to start a new company in months succeeding the interview, approximately 1.5 year after diagnosis
11	Operator (machine)	≥ 5	Perm	40	Same	Perm	40/40	• Stopped working after diagnosis, initial RTW ~ 1.5 year later. Full-time available for work at the time of the interview, but was at least partly sick-listed • Less satisfying job tasks, with perspective to return to old tasks in future
12	Health care professional	≥ 5	Perm	24	Same	Perm	24/24	• Stopped working after diagnosis, initial RTW ~ 1 year later and full RTW ~ 1.5 year after diagnosis
13	Educational professional	≥ 5	Perm	40	Same	Perm	40/40	• Stopped working after diagnosis, initial RTW ~ 10 months later
14	Self-employed	Unknown	–	50	Disability pension^a^/self-employed	NA	Unknown	• Stopped working after diagnosis/during treatment, initial RTW ~ 3.5 years later • Family members took over/continued job tasks/hours
15	Advisor	≥ 5	Perm	32	Same	Perm	32/32	• Worked until start of treatment, and performed some work tasks until hospitalization for the transplantation • Initial RTW ~ 2.5 month after SCT, ~ 8 months after diagnosis. Full RTW between 1.5 and 2 years after diagnosis

*Abbreviations*: *Pt* patient, *Perm* permanent employment contract, *Temp* temporarily employment contract, *RTW* return to work

^a^Private insurance

### RTW process

All patients were sick-listed during the treatment period or part of it (Table 2). Two patients did not RTW; one patient was already on sick-leave before cancer diagnosis and was assessed for a disability pension shortly after HSCT, and for one patient, the employment contract was not extended. The RTW process of the remaining 13 patients frequently took months or years following treatment, was often characterized by a gradual RTW in hours, workload, workplace tasks, and/or responsibilities, and was sometimes not or only partly successful. The prolonged duration and incompleteness of the RTW process was illustrated by patient 2 (quote 1, Table 3).

**Table 3:** Quotes of the patients that illustrated their work perceptions and the barriers to and facilitators for RTW they encountered after HSCT

Quote Nr.	Pt	Theme	Quote phrase
1.	2	Return to work progress	“*Well, I – uh, let me see – started in January 2012 with two times a week for four hours, reviewing every three months, adding two hours every time. The goal was to reach four days by the end of this year, but that’s no longer feasible. I stopped at three days - full days – and that has already started to become problematic.*”
2	5	Work perceptions	“*… Look, in theory, if you’ve been this ill, you’d be better off writing a novel, or just follow your childhood dreams. Or, at least, that aspect is almost a contingency to being that ill. However, I realized all I wanted to do was simply go back to work.* *…* *Would my life have become hopeless if I wouldn’t have been able to go back to work – well maybe that is a bit of an overstatement – but I do think work is important, probably more so now than I would been inclined to say before I fell ill.*”
3	2	Work perceptions	“*Previously, you had to do everything, and I’d do it all rather quickly. Now, that’s not possible anymore. So, under those circumstances I do not know... how am I supposed to get back to work? How? How can I, you know, I will never be able to manage it all. Now and again, I still consider things, but jeez.*”
4	15	Looking after one’s health—facilitator—activities and participation	*Q:* “*And the exercising at ‘Herstel en Balans’ [rehabilitation program]. Has that helped you a little in returning to work?*” *R:* “*A little? A lot! Without that – It would have taken me another year. You really do need it. I am not much of an athlete, so my goodness did I have an obstacle to surmount. But I still exercise now, because I have noticed, I just have to keep doing it.*”
5	14	Barrier—personal factors	“*Previously, it didn’t matter to me at all, you just washed your hands and were done with it, but now, now you think “oh no, I’ve got a tiny cut, should I touch that.” You know, before you know it, you’ve got another infection, which will take forever to go away.*”
6	12	Barrier—personal factors	“*Especially as a layman, if your whole life has been – tralala, tralalie – void of such things and suddenly you’re confronted with such bureaucracy. And if you have to read those forms, and – of course - they contain loads of things, which you actually don’t want to know at all. And then you think: “Gee, what am I being confronted with, while I’ve been ill and haven’t asked for this at all.” That’s just what I thought, I found it overwhelming.*”
7	15	Barrier—environmental factors—directly related to work	“*I had allocated my work among many colleagues. And they enjoyed it. And they all said “Well, but this is actually part of my job now.” So I thought hold on, hold on, what is this? Yeah, or they would just stay in a working group, or if they were asked a question they would just answer to it. They didn’t say “well, [respondent] has returned to her duties, you should take this to her.*”
8	5	Barrier—environmental factors-directly related to work	“*...You’re also going to shake your respondent’s hand. Well, those are things you cannot have at all, because I was really vulnerable. So, that was difficult. And to explain this every single time, and every time you’re like, well, I am not going to shake your hand.*”
9	12	Facilitator—factors directly related to work	“*Well, this occupational physician, − uh – I have actually set up a schedule together with him to see what is manageable and desirable? That has helped me a lot.*”
10	3	Solutions for RTW barriers	“*Especially in the beginning, because you are suddenly unemployed and you don’t know what is going to happen and you don’t know what your options and legal rights are. So, I would have appreciated it if I could have talked about that some more, or ask some questions.*”
11	2	Need for improved information	“*But I also tried appealing to the UVW [Employee Insurance Agency], like so, how will things develop in the coming period? Like, will my income decrease because of my illness? What are you going to top up, and for how long? Because if the UVW [Employee Insurance Agency], goes: “well, Mister [name of patient], your salary at the moment of being ill, well now that you’re going back to work and your salary is lower, you have a loss of revenue because of being ill. Well, we’re going to compensate for that. Well, then you won’t hear me complaining. But I’ve already tried to contact them a few times, but they’ll simply reply by telling me “well Mister [name of patient], you do not have priority right now. You’ve been scheduled in December. Then I’ll say that I am aware of that, but I would like to have clarity for myself. Because the conversations are going to happen soon, but I want to know what I can expect.*”

*Abbreviations*: *Nr* number, *Pt* patient

### Work perceptions

Most patients were positive about working and were motivated to RTW. Some patients reported that work had declined in importance, some reported that their work remained equally important, and one patient indicated that he now better realized how important work was for him (quote 2, Table 3). RTW was both associated with positive (e.g., enjoying the work task) and negative feelings (e.g., not being taken seriously, not wanting to talk about the disease). Often, patients mentioned diminished work ability (Table 4). Patient 2 illustrated his struggle with the inability to perform his old job tasks (quote 3, Table 3).

**Table 4:** Work perceptions of the 15 HSCT survivors that participated in our study

RTW motivations • Not wanting to be at home/getting out and about • Wanting distraction • Live up to expectations of society • Not wanting to be a patient anymore • Returning to work as proof of recovery • Having positive work ethos: ○ Not wanting to be on benefits ○ Feeling commitment toward society at large • Not wanting to run into financial difficulties	Work values • Work as a goal in life • Mean something • Return to society • Altered work values: ○ Health and family more important ○ Resignation about work future ○ Less easily concerned about work future
Perceived work ability • Being able to meet work demands • Being realistic about own work ability • Experiencing not being able to/having trouble with meeting work demands due to: ○ Recovery time needed/interference with social/family life ○ Long-term side effects of diagnosis and treatment • Being uncertain about own work ability	Feelings about (not being able) (to return to) work • Acceptance of health limitations that interfere with (being able to) work • Enjoy working • Negatively altered relationships at work • Being officially work disabled felt as being of no account • Returning to work felt as a lonely process

### Barriers to and facilitators of RTW

All patients reported several barriers to and facilitators of RTW across different ICF-domains (Table 5). Many factors were identified both as barrier to some patients and as facilitator to others, mainly depending on the direction of the factor (e.g., support being absent or present).

**Table 5:** Facilitators for and barriers to RTW categorized according to the ICF

ICF domain	ICF factor	Barrier (−)/facilitator (+)
Disease/disorder and body functions, body structure	(Duration of) cancer treatment and appointments at outpatient clinic	–
	(Absence of) long-term side effects of hematologic cancer treatment and medication	−/+
	Temperament and personality functions	−/+
	Comorbidity and pre-diagnosis poor health	–
Activity/participation	Looking after one’s health	+
	(Having difficulties) with economic self-sufficiency	−/+
	Having difficulties commuting	–
	Having difficulties with doing household tasks including taking care of children	–
Personal factors	(Not) having adequate skills/competences	−/+
	(Not) having difficulties coping	−/+
	(Negatively altered) importance of work	−/+
Environmental factors—directly related to work	Negative/positive job content	−/+
	Negative/positive work conditions	−/+
	Negative/positive terms of employment	−/+
	(Absence of) support and understanding supervisor	−/+
	(Absence of) support and understanding colleagues	−/+
	(Absence of) support and understanding occupational physician	−/+
	(Absence of) support and understanding human resource management	−/+
	Support and understanding customers	+
	Keeping in touch with workplace	+
Environmental factors—not directly related to work	(Absence of) support and relationship—health professionals	−/+
	Support and relationship—former cancer patients	+
	Support and relationship—immediate family	+
	Service systems and policies	−/+

#### Body functions and structure

Identified RTW barriers included impairments resulting from the long duration of cancer treatment and the slow or insufficient recovery of the physical (e.g., fatigue, weakened immune system) and mental (i.e., cognitive functioning) impact of the disease and treatment. In addition, temperament and personality functions (e.g., being too tough for oneself) were mentioned as barriers. Absence or recovery of side effects and temperament and personality functions (e.g., having confidence, being independent) were identified as RTW facilitators.

#### Activities and participation

Taking care of household tasks and/or children, and commuting were mentioned as barriers. Perceived facilitators included ‘fear of losing his/her job,’ or ‘looking after one’s health’ (e.g., using psychotherapy or participating in a rehabilitation program or sports), see Table 3, quote 4.

#### Personal factors

A wide variety of personal barriers to and facilitators of RTW were identified, which can be divided into the following domains: the importance of work, skills/competences (e.g., having difficulties with the work load) and coping strategies (e.g., having difficulties with accepting limitations, having difficulties with performing job tasks due to fear of infection (quote 5, Table 3), and having difficulties with RTW insecurity (quote 6, Table 3)).

Personal RTW facilitators included ‘gaining required information’ (skills/competences), ‘discuss perceived limitations with supervisor/colleagues,’ and ‘take the initiative to plan RTW’ (both coping strategies).

#### Environmental factors—directly related to work

Specific characteristics of the job content (e.g., only being able to do the job full-time) and working conditions (e.g., not having their workplace anymore) were mentioned as barriers. In addition, three negative aspects of employment were pointed out: being fired due to long-term sick-leave, being self-employed, and difficulties getting back to the previously held position (quote 7, Table 3).

In addition, either already existing negative work relations or negatively altered relations at the workplace due to cancer were mentioned as RTW barriers. Patients mentioned that supervisors and/or colleagues found it difficult to deal with “cancer” at the workplace, that they experienced insufficient recognition from colleagues, no understanding about the (long-term) consequences of HSCT, disagreement about which tasks the patients were entitled to do, and they experienced difficult encounters with customers. For instance, patient 5 often had to shake hands with business relations, but was now unable to do so because of the risk of infections (quote 8, Table 3).

Job content (e.g., not having physically heavy work) and work conditions (e.g., being able to work from home) and aspects of employment (e.g., being able to gradually RTW, flexibility in working hours, being able to (temporarily) do less demanding tasks) were also mentioned as facilitators. The importance of keeping in touch with the supervisor and/or colleagues was emphasized. Work relations provided support and understanding and a safe place to return to. Adequate RTW support from the supervisor, occupational physician, and human resource management was also identified as facilitator. Patient 12 reported receiving autonomy from a supervisor and the influence of the occupational physician on RTW (quote 9, Table 3).

#### Environmental factors—not directly related to work

‘The lack of RTW support from the hospital’ was mentioned as a barrier, while support from healthcare professionals, other cancer survivors, and immediate family members were reported as RTW facilitators. The rules and regulations were both frequently mentioned as barriers and facilitators. For some patients, the fear of losing their job and/or the financial consequences of being on extended sick-leave urged them to RTW. In one patient, however, this incentive made her return too quickly, causing a (worse) recurrent sick-leave.

### Solutions to improve RTW

Patients often found it difficult to identify solutions for RTW barriers. Some patients mentioned that they would have liked to talk about work issues at the hospital (quote 10, Table 3) and others would have liked more support from their employer (longer period in which the patient receives 100% of their original salary, regular meetings to discuss RTW progress). Also, improving the opportunities to participate in a rehabilitation or physical exercise program was mentioned. Many patients reported the need for information about the financial consequences of being on sick-leave, long-term disability, and the rights and rules when sick-listed (quote 11, Table 3).

## Discussion

This qualitative study explored work perceptions, barriers to, and facilitators of RTW, and possible solutions to improve RTW among HSCT survivors. At a mean 2.5 years after HSCT, approximately half of the patients had not or only partially returned to work. Most HSCT survivors valued work as positive and they were motivated to RTW. Still, RTW was often a long-lasting process associated with many perceived uncertainties about RTW and the associated financial aspects. These findings indicate the need for RTW support for HSCT survivors before, during, and after initial RTW.

### Work perceptions

Most patients were very motivated to RTW, and for many patients work was an important topic in their lives. Despite the distinctive aggressiveness of the HSCT and the higher risks on medical complications and symptoms when compared to other cancer treatments [2], these and the other mentioned work perceptions were comparable to those found in meta-syntheses in which the qualitative literature on the experiences with work of survivors of various types of cancer had been reviewed [15, 16]. Consequently, work perceptions seem to be generalizable across different populations of cancer patients.

Despite the similarities across populations, work perceptions differed considerably at the individual level. For instance, for some patients, the positive feelings about work stood out, while for others negative feelings were evident. Tiedtke et al. [26] suggested that different experiences likely influence what type of support is warranted. Therefore, when advising patients on RTW, and/or when advising health care professionals, supervisors, or colleagues on how they can support an employee/colleague diagnosed with cancer, it appears to be more important to focus on individual work perceptions, rather than assuming differences in work perceptions based on their cancer type and/or treatment.

### Barriers to and facilitators of RTW and possible solutions to improve RTW

The patients in our study were unable to work during at least a part of the treatment, and it often took months to (partially) RTW. This can be explained by the distinct aggressiveness of this type of treatment and its frequent medical complications (e.g., infections and/or graft-versus-host-disease). Our study corroborates findings from previous studies among patients treated for a hematologic malignancy that ongoing physical and psychological problems are barriers to RTW [17, 18]. It furthermore corroborates findings from employers’ and survivors’ perspective that support of the employer and of colleagues, as well as flexible working hours and work tasks/conditions are important facilitators of RTW [27, 28]. Similarly, it supports previous findings among cancer patients that report on the importance of personal factors such as coping and self-efficacy [29]. In addition, and in line with previous studies [15–18], the financial impact of being sick-listed was important for our patients. The (fear of the) financial consequences of being sick-listed and being assessed for a disability pension were experienced as RTW facilitator. Although the existence of a financial incentive to RTW might be positive for some patients, our study showed that it becomes problematic when patients are forced to RTW while they are physically and/or psychologically unable to perform the job tasks, posing the patients at risk for recurrent sick-leave. Unfortunately, this issue is difficult to overcome, as it is a consequence of national laws and regulations.

There were some discrepancies between our findings and those reported previously. First, in contrast to a previous qualitative study among patients with hematologic malignancies [18], our patients did not mention approval for RTW provided by the hematologist as a facilitator for RTW. However, talking about work issues in the hospital and improved support and information about sick-leave, disability pension and RTW were identified as potential solutions to improve RTW. Tamminga et al. [30] did not find favorable effects of a hospital-based work support intervention when compared to usual care on RTW outcomes, QoL, and work ability among 133 breast and gynecological cancer patients, although the intervention was considered useful by the intervention providers (nurses) and was appreciated by patients. It is yet unclear whether addressing RTW barriers by the hematologist, specialist nurse, or social worker would improve RTW of HSCT survivors.

Second, inconsistent with the findings of previous studies [15–17], our patients did not mention problems with disclosing the diagnosis to colleagues and employers. It could be that the patients had no other option: The small timeframe between diagnosis and start of treatment and/or the required hospital admission might have forced patients to disclose the diagnosis. Still, also the Dutch social security system in which employees are protected against dismissal due to illness might play a role [21]. In four other Dutch qualitative studies on work experiences of in total 72 cancer patients (breast cancer: *n* = 41) [21, 31–33], only one study reported that some patients did not want to disclose their disease to colleagues [31].

Third, the perceived importance of looking after one’s health, and specifically the participation in rehabilitation programs or sports as facilitator for RTW have not been reported previously in patients with hematologic malignancies [17, 18], nor in the previous reviews [15, 16], but had been mentioned earlier by Dutch cancer survivors [21, 33]. Additionally, possible positive effects of exercise on RTW have been reported in qualitative studies among cancer patients who had participated in an exercise program [32, 34]. Currently, however, only a few RCTs have evaluated the effects of a physical exercise/activity intervention on RTW, with conflicting results [35, 36]. In their Cochrane review, De Boer et al. [37] found moderate quality evidence that interventions in which vocational counseling is combined with patient education, patient counseling, and biofeedback-assisted behavioral training or physical exercise, result in higher RTW rates. Further rehabilitation trials might therefore consider including vocational counseling in their intervention program and to include RTW as secondary outcome measurement.

### Strengths and limitations

A strength of this study is the inclusion of a heterogeneous study population in terms of pre-diagnosis working situation, RTW trajectories, and physical health. This provides a broad perspective of the barriers to and facilitators of RTW that HSCT survivors perceive. Furthermore, the use of themes from an overview of issues that might contribute to the quality of working life of employees with a chronic disease [23] and the ICF model [24] resulted in a structured overview of the results and allows comparison with other studies. A limitation of this qualitative study is the fact that we did not check for data saturation. Therefore, it might be that saturation was not reached, and additional interviews could have resulted in new themes. However, our results could be used as a starting point for the development of questionnaires, studying (return to) work perceptions, and barriers to and facilitators of RTW of HCTS survivors, which could be administered to a large and representative sample. Furthermore, in the fourth step of the analysis (i.e., when open codes were classified into the ICF model), we used a deductive coding technique. It is possible that a different classification of barriers to and facilitators of RTW had emerged, if we had developed a new model based on our open codes. However, the use of existing models enables comparison with other studies and resulted in a structured overview.

Another limitation is the fact that there is still a need for a prospective cohort study to determine which of the perceived barriers and facilitators are actual predictors of RTW outcomes and to determine which of the suggested solutions to improve RTW might actually improve RTW. Non-amendable predictors (e.g., age) could provide us insight which population is at risk of adverse RTW outcomes. Modifiable predictors (e.g., employer support) and suggested solutions to improve RTW could yield insight into which factors should be targeted by an intervention program or policy, or what the content of such an intervention program or policy should be.

In conclusion, this qualitative study showed that many HSCT survivors value work as positive and they are motivated to RTW. Still, RTW was often a lengthy and complex process. Insight in work perceptions, RTW barriers, and solutions might help researchers, healthcare professionals, and employers to develop and/or tailor individualized multidisciplinary care to facilitate RTW.
